# A histological method for quantifying *Plasmodium falciparum* in the brain in fatal paediatric cerebral malaria

**DOI:** 10.1186/1475-2875-12-191

**Published:** 2013-06-07

**Authors:** Danny A Milner, Clarissa Valim, Richard A Carr, Pankaj B Chandak, Nedson G Fosiko, Richard Whitten, Krupa B Playforth, Karl B Seydel, Steve Kamiza, Malcolm E Molyneux, Terrie E Taylor

**Affiliations:** 1 Anatomic and Clinical Pathology, Brigham and Women’s Hospital, Boston, MA, USA; 2 Immunology and Infectious Diseases, Harvard School of Public Health, Boston, MA 02115, USA; 3 Anatomic Pathology, Warwick Hospital, Warwickshire, UK; 4 Surgery, Guy’s and St. Thomas’ Hospital, London, UK; 5 Histopathology, University of Malawi College of Medicine, Blantyre, Malawi, Africa; 6 Anatomic and Clinical Pathology, Cellnetix Pathology and Laboratories, Olympia, WA, USA; 7 Psychology, Harvard University, Cambridge, MA, USA; 8 Medicine, College of Osteopathic Medicine, Michigan State University, East Lansing, Michigan, USA; 9 Malawi-Liverpool-Wellcome Trust Clinical Research Programme, Blantyre, Malawi

**Keywords:** Cerebral Malaria, Africa, Paediatric, Autopsy, Quantification, Histology, Plasmodium Falciparum

## Abstract

**Background:**

The sequestration of *Plasmodium falciparum*-infected erythrocytes in brain microvasculature through cytoadherence to endothelium, is the hallmark of the definitive diagnosis of cerebral malaria and plays a critical role in malaria pathogenesis. The complex pathophysiology, which leads each patient to the final outcome of cerebral malaria, is multifaceted and thus, metrics to delineate specific patterns within cerebral malaria are needed to further parse patients.

**Methods:**

A method was developed for quantification utilizing counts of capillary contents (early-stage parasites, late-stage parasites and fibrin) from histological preparations of brain tissue after death, and compared it to the standard approach, in which the percentage of parasitized vessels in cross-section is determined.

**Results:**

Within the initial cohort of 50 patients, two different observers agreed closely on the percentage of vessels parasitized, pigmented parasites and pigment globules (ICC = 0.795-0.970). Correlations between observers for correct diagnostic classification were high (Kendall’s tau-b = 0.8779, Kappa = 0.8413). When these methods were applied prospectively to a second set of 50 autopsy samples, they revealed a heterogeneous distribution of sequestered parasites in the brain with pigmented parasites and pigment globules present in the cerebellum > cortex > brainstem. There was no difference in the distribution of early stages of parasites or in the percentage of vessels parasitized across the same sites. The second cohort of cases was also used to test a previously published classification and regression tree (CART) analysis; the quantitative data alone were able to accurately classify and distinguish cerebral malaria from non-cerebral malaria. Classification errors occurred within a subclassification of cerebral malaria (CM1 *vs* CM2). A repeat CART analysis for the second cohort generated slightly different classification rules with more accurate subclassification, although misclassification still occurred.

**Conclusions:**

The traditional measure of parasite sequestration in falciparum malaria, the percentage of vessels parasitized, is the most reliable and consistent for the general diagnosis of cerebral malaria. Methods that involve quantitative measures of different life cycle stages are useful for distinguishing patterns within the cerebral malaria population; these subclassifications may be important for studies of disease pathogenesis and ancillary treatment.

## Background

Five species of malaria parasite can infect humans, but one, *Plasmodium falciparum*, is responsible for nearly all of the severe morbidity and mortality in malaria-endemic areas [1]. A distinguishing feature of *P*. *falciparum* infections is the ability of infected erythrocytes, especially those containing parasites in the latter half of their life cycle, to adhere to the endothelium (i.e. cytoadherence) of capillaries and post-capillary venules [2]. This process leads to the accumulation of parasites in tissues, a phenomenon known as sequestration, which is so effective that mature, pigmented forms of *P*. *falciparum* are rarely seen in the peripheral blood [3-6]. Eventually, erythrocytes containing mature parasites (schizonts) rupture, releasing merozoites and malaria pigment, haemozoin. This malaria pigment is the remnant of the parasite-driven process of haemoglobin degradation and is scavenged and removed by monocytes.

The clinical syndromes associated with *P*. *falciparum* infections range from asymptomatic parasitaemia to convulsions, coma and death. The mechanisms leading to central nervous system dysfunction in severe malaria have not been elucidated, but one hypothesis suggests that sequestration of parasitized erythrocytes in cerebral microvessels with resultant vascular congestion plays a crucial part in pathogenesis [7,8]. To gain further insight into this hypothesis, it is necessary to have a standardized approach to assess cerebral tissue with respect to malaria findings.

A clinicopathological study of fatal cerebral malaria is ongoing in Blantyre, Malawi. A reproducible and biologically relevant method of quantifying sequestration is necessary before comparisons between sites within an organ, or between patients, can be made. The traditional method is to establish, in a given tissue sample, the proportion of vessels which contain parasitized erythrocytes. This figure is then used as the basis for comparisons [9-13]. This approach was expanded this approach to include measures of the intensity of and life-cycle stage of the sequestered parasites, using histological preparations of brain tissue. The two approaches are described and compared is this study. To evaluate the biological utility of the measures, new cohort of patients was used to test a classification system based on a previous CART analysis [14]. A new CART analysis using a second cohort of patients was performed.

## Methods

### Patients

All patients were admitted to the Paediatric Research Ward (Queen Elizabeth Central Hospital, Blantyre, Malawi) between February 1996 and June 2010. Demographic, historical, clinical, and laboratory information were recorded for the duration of admission. The Blantyre Coma Score [14] was used to estimate severity of coma. Lumbar punctures to rule out meningitis were performed on all children with altered consciousness (Blantyre Coma Score <5) unless there was a clinical contra-indication. Following diagnosis, children were managed according to standard protocols as previously described [15]. Clinical diagnoses were determined at the time of discharge or death. Patients were assigned a clinical diagnosis of cerebral malaria (Clin CM) if they were admitted with coma (a Blantyre Coma Score ≤2 lasting for more than two hours after admission with no evidence of meningitis), *P*. *falciparum* parasitaemia, and no improvement following effective treatment for seizures and hypoglycaemia. Comatose children who were aparasitaemic on four consecutive blood smears collected at six-hourly intervals were diagnosed as having non-malarial comas (NM Coma). Parasitaemic children without coma (Blantyre Coma Score >2 within two hours of admission) and a haematocrit <15% were classified as having severe malarial anaemia (SMA). Parasitaemic children without coma (Blantyre Coma Score >2 within two hours of admission) were classified as having other malarial illness (other). Parasitaemic children who died before a clinical diagnosis could be determined were grouped together as indeterminate. Parasitaemic children with an obvious non-malarial cause of coma were classified as non-malarial coma with incidental parasitaemia (NM Coma IP). All were treated initially with quinine; blood and antibiotics were administered according to standard criteria, and the treatment regimen was adjusted as the working diagnosis evolved [15]. The study was approved by the ethics committees at the University of Liverpool, Michigan State University, and the University of Malaŵi College Of Medicine.

### Autopsies

In the event of a death, a Malaŵian clinician met with key family members to discuss the possibility of performing an autopsy. When permission was granted, autopsies were performed in the mortuary of the Queen Elizabeth Central Hospital. The brain was cut directly at autopsy and blocks from standard regions were fixed in 10% pH neutral buffered formalin, processed per routine protocol, and embedded in paraffin. Sections cut at 5 μ were stained with haematoxylin and eosin.

### Pathological classification

Histological sections of brain tissue were reviewed by pathologists (RAC, DM, SK and RW) who were blinded to the clinical diagnosis and were classified as follows: sequestration only (CM1); sequestration and extravascular pathology including ring haemorrhages (CM2); and no evidence of sequestration/malarial pathology (CM3). The brain and remaining organs were evaluated systematically to determine cause of death.

### Quantitative method training

**Figure 1:**
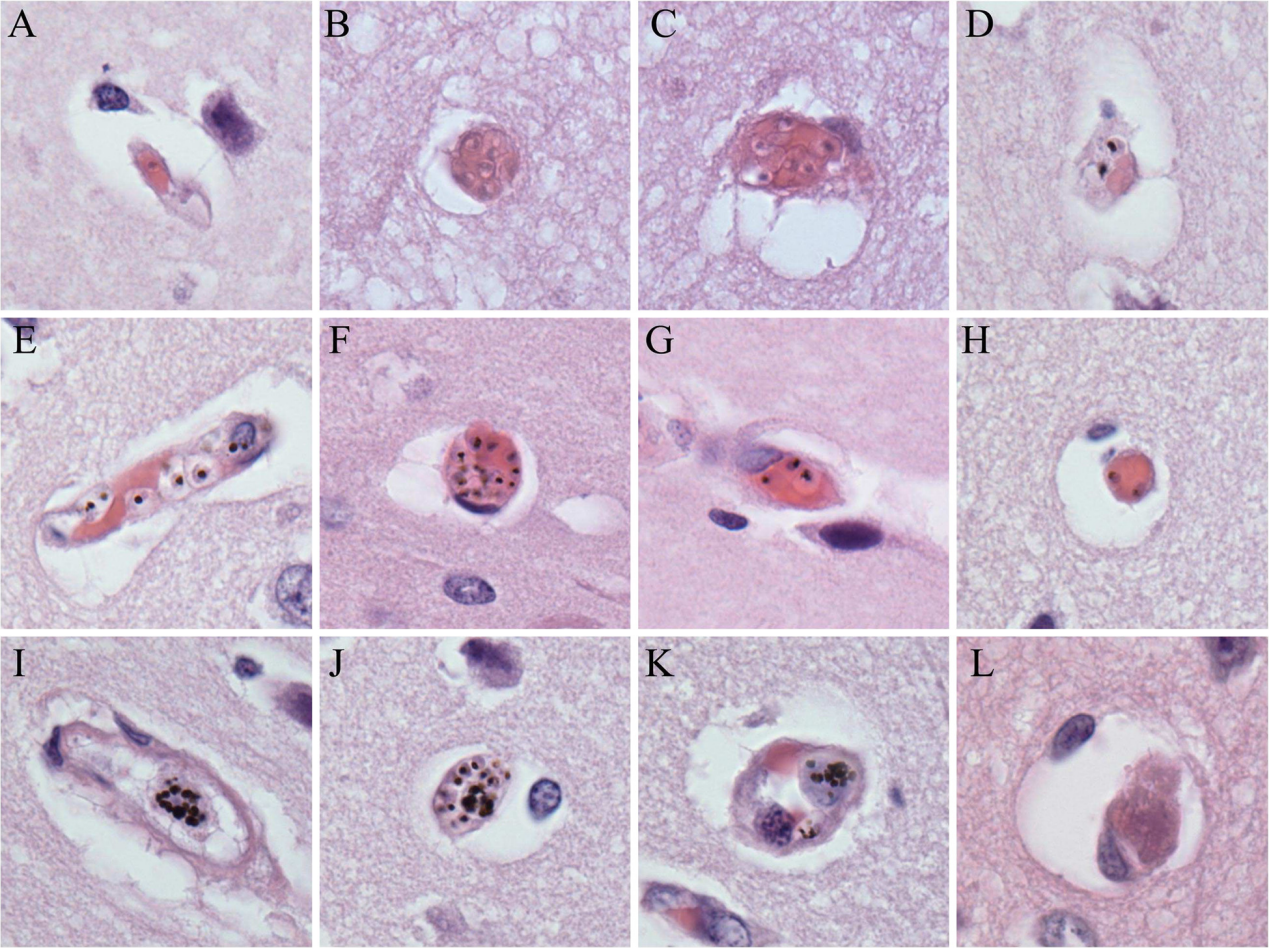
**Examples of capillary contents.** Note that only the capillaries in Panels **B**, **D**, **F**, **H**, **J**, **K**, and **L** would be adequate for counting by our method; the other panels are illustrative of parasite elements but would not be used for counting. All images haematoxylin and eosin at 1,000× original magnification.

Fourteen cases with high rates of parasite sequestration (>50% of capillaries parasitized) in the cerebral microvasculature were included. Each was coded and an area was outlined on each slide where the grey matter was sectioned perpendicularly to the meningeal surface of the brain. The slide was secured on a standard microscope such that the outlined area of grey matter ran perpendicularly to the up/down or side/side direction of the microscope stage. Consecutive fields were counted, perpendicular to the meningeal surface, running toward the border between grey and white matter. At the completion of a run, the slide was moved across two field-widths followed by a second run back up to the meningeal surface. Microscope fields were assessed systematically until at least 100 cross-sectioned capillaries were identified and a run was complete (see Additional file 1 for counting method tutorial). Capillaries (Figure 1) were defined as a circular or oval blood vessel profile with a maximum-to-minimum diameter of ≤2:1 and having at most one visible endothelial cell nucleus in the wall. Examples of the vessels encountered in a routine section are shown in Figure 1 and include the following: (A) A single unpigmented parasites in a vessel; (B) a cluster of unpigmented parasites within a capillary; (C) a cluster of unpigmented parasites in an elongated vessel; (D) two pigmented parasites in a capillary; (E) several pigmented parasites in a longitudinal vessel; (F) a large clustered of pigmented parasites in a capillary; (G) three pigmented parasites in a longitudinal vessel; (H) two pigmented parasites in a capillary; (I) a white blood cell containing many globules of pigment within a longitudinal vessel; (J) a white blood cell containing many globules of pigment within a capillary; (K) a white blood cell with pigment within a dilated capillary; (L) a capillary filled with fibrin without evidence of ring haemorrhage. Individual elements were quantified as follows:

**unpigmented parasites** (**UPP**): Parasites were counted as unpigmented if no pigment was seen within the erythrocyte and if the parasite generally occupied less than one third of the diameter of the erythrocyte (Figure 1B and 1C);

**pigmented parasites** (**PP**): The common feature in these parasitized erythrocytes is the presence of malarial pigment which can vary in size from a small black dot to a less well defined larger mass of brown/black pigment (Figure 1D-1H, 1J);

**extra**-**erythrocytic malaria pigment** (**PG**): Malaria pigment appears as a single mass of ill-defined black pigment associated with a ‘ghost’ red blood cell, free in the vessel, or as multiple masses within intravascular white blood cells (Figure 1G, 1I-1K);

**the traditional measure** (**percentage of cross**-**sectioned vessels containing intact parasites** (%**VP**); **and fibrin**: Identified on hematoxylin and eosin stain and quantified per 100 capillaries (Figure 1L).

Counts were performed twice by an experienced observer (RAC), and twice by three other individuals (CP, DM, and NF), following instruction by, and under the supervision of the experienced observer. All counts were made without knowledge of the results of other readings.

### Quantitative method study

Slides from the first 50 autopsies (MP1 to 50) of the series were prepared from the parietal lobe, midbrain, and cerebellum and the various parasite elements were counted as described above by a single observer (NF). For the parietal lobe, a second observer (DM) counted all parasite elements for all 50 cases. Modifications of the method were required in the occipital grey matter (data not utilized in this manuscript) where the runs were completed to the meningeal surface of white matter/grey matter junction when a minimum of 100 vessels had been assessed. In order to represent different layers within each region of the brainstem equally, areas on the para-sagittal plane of each slide were delineated, running in the longest possible axis. Runs were completed from the top to the bottom of the area to include a minimum of 100 vessels. In the cerebellum, an area of the slide was chosen in which the cerebellar folia were well represented, and consecutive fields were counted, running within the molecular layer of the folia until 100 vessels were examined. The results of this quantification (for NF only) were previously analysed using CART and published [15].

### Brain distribution study

For a subset of 28 of the first 50 cases, counts were performed on the parietal lobe, occipital lobe, midbrain, pons, medulla, and cerebellum by one observer (CP).

### CART analysis (validation and repeat)

Slides from the second 50 cases of the series (MP51 to 100) were prepared from one area of the cortex (parietal lobe). The various parasite elements were counted as described by a single observer (DM). Counts were performed blinded to the clinical and pathological diagnoses.

## Statistics

### Reliability in quantification of parasite elements

The complete counts for the first 50 cases (regardless of clinical and pathological diagnosis) were compared between two observers (NF and DM) for each element by analysis of agreement (Bland-Altman plot), reproducibility (intraclass correlation co-efficients with 95% confidence intervals), and correlation (visualization of scatter plots and Spearman correlation co-efficients). Regression analysis for each variable was also performed. Additionally agreement for case classification as based on clinical and histopathological diagnosis of each observer was assessed via weighted Kappa co-efficients with corresponding P-values. Classification of the 50 cases was performed through %VP with cut-offs and rule defined by the previous CART analysis. For each observer, the cases were classified by the actual counts. Distribution of each element between the two observers was compared based on means and t-tests with p-values adjusted for multiple testing via Bonferroni approach (0.05/70).

### CART analysis (validation and repeat)

All counted parasite elements were normalized to 100 vessels for all patients and the following variables were used as potential node differentiators:- UPP, PP, PG, TPG, fibrin, and %VP. For the purposes of clinical validity, all patients who met the clinical case definition of cerebral malaria were included as categories for classification, which included CM1, CM2, and CM3 (i.e. non-CM patients were excluded). Validation of the previous CART analysis was performed by classifying the latter 50 cases with the rules established by the first 50 cases. Briefly, the rule identified by the previous CART analysis classified as true CM (CM1 or CM2) patients who had greater than 23% of vessels parasitized, CM2 patients as those with greater than 55.5 pigment globules per 100 vessels, and as non-CM (CM3) patients as those less than 25% of vessels parasitized. A repeat *de novo* CART analysis was performed using only the latter 50 cases and this method was run with five times cross-validation. CART software (Salford Systems, San Diego, CA, USA) was used for all tree analysis.

## Results

### Patients

Data from 100 patients were included; 71 patients met the clinical case definition of cerebral malaria of which 13 were pathologically classified as CM1, 39 as CM2, and 19 as CM3. The remaining 29 patients included four patients with SMA, 21 with non-malaria causes of coma or other non-malarial illnesses, and four indeterminate cases (the patients died before a clinical diagnosis could be determined).

### Reliability of quantification method

A Bland-Altman plot of %VP was performed first and demonstrated excellent agreement between observers (Additional file 2). The bias or difference in individual counts between the two observers was generally 20 or lower (within two standard deviations). The intraclass correlation co-efficient (ICC) also showed very good to excellent reproducibility of counting for most individual elements (0.82-0.97), except for fibrin (0.284) and UPP (0.314) (Table 1). Expert review of discrepant cases showed that one observer systematically undercounted UPP and overcounted fibrin. Visualization of the scatter plots was consistent with reproducibility analysis, demonstrating the strongest correlations between percentage of vessels parasitized, total parasites per 100 capillaries, and pigment measurements (Additional file 3). Classification of cases based on the counts generated by either observer using the CART rules was accurate with Kappa scores >0.95 (Table 2). The mean of the individual parasite elements, as counted by both observers across diagnostic categories, demonstrated significant differences only in counts of fibrin and pigment globules (p < 0.0009 by Bonferroni) (Additional file 4).

**Table 1:** **Intraclass correlation co**-**efficient**

**Variable**	**Intraclass**	**95% CI**	
	**Correlation**		
	**Coefficient**		
% Vessels Parasitized (vpc)	0.970	0.948	0.983
Unpigmented Parasites (upp)	0.314	0.039	0.545
Pigmented Parasites (pp)	0.823	0.706	0.900
Pigment (pg)	0.905	0.837	0.945
Total Pigment (tpg)	0.928	0.876	0.959
Fibrin	0.284	0.006	0.522

Intraclass correlation co-efficient (CV) for two observers (NF and DM) comparing counts for the first 50 cases by each element.

**Table 2:** Comparison of the clinicopathological diagnosis with the histological count classification

**Comparison**	Agreement****	Kappa*	**P**-**value**
CM** vs. NF***	97.55%	0.9532	<0.0001
CM vs. DM***	97.96%	0.9612	<0.0001
NF vs. DM	96.33%	0.9302	<0.0001

*Weighted kappa based on [1, 0.8 1, 0 0 1] matrix.

**The CM classification is based on the clinical data (whether or not the patient met the clinical case definition of cerebral malaria) plus the pathological data in three categories (1 = sequestration only, 2 = sequestration plus extravascular pathology, 3 = non-malaria cause of death). For the purposes of this analysis, all malaria pathology negative patients (including those that met the clinical case definition but died of another cause) are categorized as 3. Two early cases who died very quickly without proper clinical assessment are classified as 1 for this analysis based solely on pathology.

*** For both NF and DM, the counts produced for the cases were used to classify the patients as 1 (if % of vessels parasitized >23.3 and total pigment globules <55.5), 2 (vessels parasitized >23.3 and total pigment >55.5) or 3 (vessels parasitized <23.3) corresponding to the outcome of the classification and regression tree analysis previously published [15].

**** Among the total 50 observations done by the two readers, proportion of those in which both observers equally classified the case.

Comparison of the clinicopathological diagnosis (clinical data plus pathological data final anatomic diagnosis) compared with the histological count classification for both NF and DM.

### Brain distribution of parasite elements

**Figure 2:**
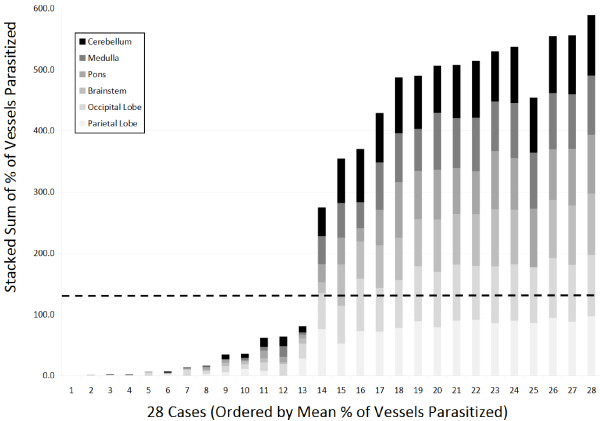
**The distribution across 28 sequential cases of vessel sequestration (as measured by % of vessels parasitized) across six sites within the brain are shown as stacked bars (maximum** **=** **600 represented 100% of vessels parasitized × 6 sites).** Cases are ordered by mean % of vessels parasitized. The dotted horizontal line represents the overall cut off for “cerebral malaria” (23.3% × 6 sites = 139.8) and demonstrates that, for total brain quantification, there is a clear cut-off between controls (non-cerebral malaria, cases 1–13) and cases (cerebral malaria, cases 14–28).

**Figure 3:**
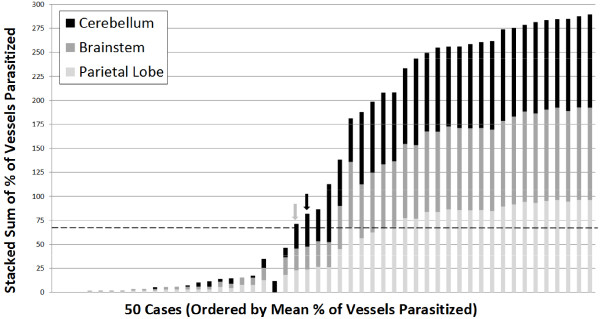
**The distribution across 50 sequential cases of vessel sequestration (as measured by % of vessels parasitized) across three sites within the brain are shown as stacked bars (maximum** **=** **300 represented 100% of vessels parasitized × 3 sites).** Cases are ordered by mean % of vessels parasitized. The dotted horizontal line represents the overall cut-off for “cerebral malaria” (23.3% × 3 sites = 69.9) and demonstrates two outliers as follows: (grey arrow) a case of coma of other cause with anaemia and no evidence of retinopathy whose parietal lobe measures was <23.3% but cerebellum was >23.3%; and (black arrow) a case of severe malaria anaemia whose parietal lobe measure was >23.3% but the child did not meet the clinical case definition of cerebral malaria.

**Figure 4:**
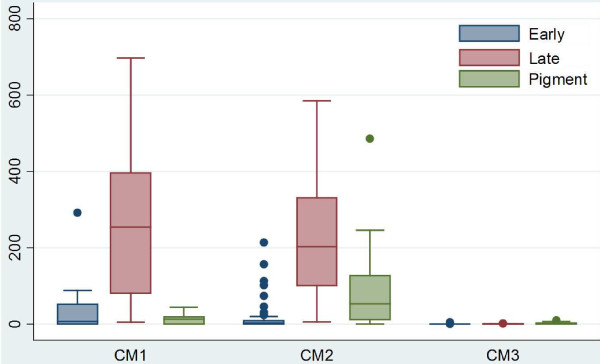
**A graphical representation of the distribution of diagnoses across parasite elements counted (early stage** **=** **unpigmented rings, late stage** **=** **pigmented trophozoites, pigment** **=** **total pigment globules) by medians demonstrating the predominance of pigment in CM2 and the primary finding of late stage parasites in most infections for the validation set (n** **=** **41).**

Distribution was consistent throughout the brain such that patients who were found to have high levels of sequestration in one site consistently had sequestration across six sites for 28 selected cases (Figure 2 and Additional file 5). Similarly, when counts in the first 50 cases were made across three sites, the distributions were consistent and demonstrated that the cerebellum was the site of highest sequestration for most cases (Figure 3 and Additional file 6), although this difference was not statistically significant. When individual parasite elements (unpigmented early parasites *vs* pigmented late parasites *vs* pigment only) were analysed across the pathological diagnoses, CM3 was significantly different (p-values <0.001, Wilcoxon Rank Sum) from CM1 and CM2 across all elements and CM2 had significantly more pigment (p-value <0.01) than CM1 (Figure 4).

### CART analysis

**Figure 5:**
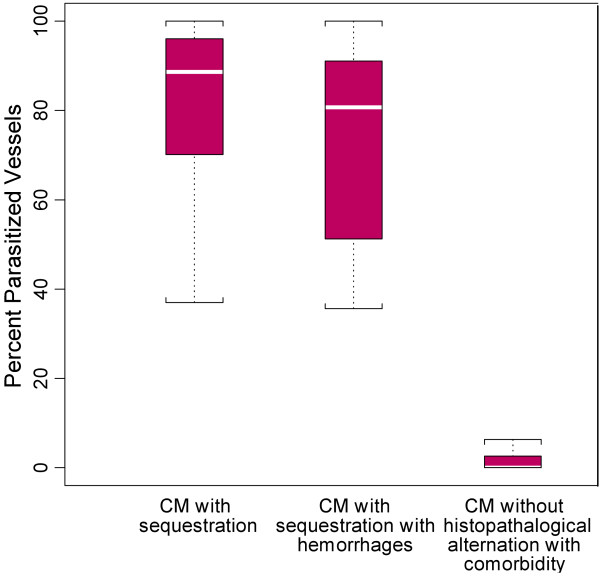
**A graphical representation of the first node of the classification tree demonstrating that the resultant cut-offs (23.3% and 21%) were stable but moot as the percentage of vessels parasitized showed no overlap when only patients meeting the clinical case definition of cerebral malaria were analysed.**

**Figure 6:**
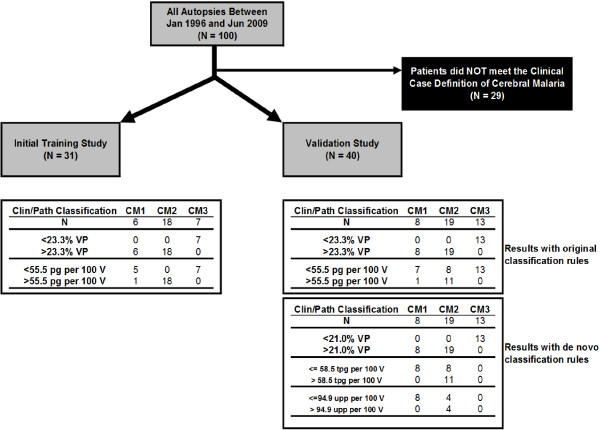
**Study flow chart and classification results from CART analysis.** The initial training and testing study (on left) represents the original data from [15]. The validation study, using 40 new cases, is shown on the right with the original classifications rules applied (upper results table) and a *de novo* set of classifications rules (based on the 40 new cases, lower results table). The first node (separating patients with any cerebral sequestration from patients without evidence of cerebral sequestration) remained stable (23.3% *vs* 21%) with no classification errors. The second node (separating patients with sequestration only from patients with sequestration and extravascular pathology including ring haemorrhages) also remained stable (58.5 pg/100 vessels *vs* 55.5 pg/100 vessels). A third node was required (<= or >94.9 UPP/100 vessels) to separate the remainder of these latter two categories; however there were, in total, four classification errors using this new model.

Using the second cohort of 50 patients, %VP perfectly distinguished subjects who had true CM from those with non-CM diagnoses (CM1 or CM2 *vs* CM3). The levels of %VP were very low for subjects with CM3 and indistinguishable from control subjects. Using the previously established cut-off level of 23.3% for %VP, the sensitivity and specificity of %VP to discriminate subjects among patients meeting the clinical case definition (i.e., “true CM” of CM1 or CM2 *vs* CM3) was 100% (Figure 5) [15]. Optimality of this cut-off level was confirmed by re-analysing the second data set using CART; this showed a nearly identical cut-off point (Figure 6). When trying to further distinguish CM in patients with sequestration only (CM1) and patients with sequestration and haemorrhages/extravascular pathology (CM2), the previously defined rule (55.5 pg/100 vessels) did not perform well. When categorizing subjects with sequestration only (CM = 1) on the basis of %VP ≥23.3% and PG/100 V <55.5, among the eight subjects with sequestration only, one was misclassified as sequestration and haemorrhage (12.5%). When categorizing subjects with sequestration and haemorrhages (CM = 2) on the basis of %VP ≥23.3% and PG/100 V ≥55.5, among the 19 subjects with sequestration and haemorrhages, eight were misclassified as sequestration only (42.1%).

A next attempt included defining a new classification rule to distinguish the three categories of clinical CM using the testing dataset. As expert review revealed that UPP and fibrin were under- and overcounted by one observer, *all* elements for the new counts by the second observer were used in the repeat CART analysis. Based on the new rule, all patients with %VP ≤21.0%, a value close to that emerging from the first CART analysis, were classified as not having CM (13/13) (Figure 6). Patients with %VP >21.0%, PG < =58.5 and UPP < =94.9 were correctly classified as patients with sequestration only (8/8). Fifteen patients were correctly classified as having sequestration with haemorrhages. However, four patients who actually had sequestration with haemorrhages were misclassified as sequestration-only based on the rulecombining %VP, PG and UPP.

## Discussion

In a series of 50 autopsies performed on American soldiers dying of clinically defined cerebral malaria during World War II, examination of haematoxylin and eosin-stained slides showed that “the entire capillary network of the brain…was distended with erythrocytes” in 46 cases [5]. The distribution of parasitized cells in the capillaries was noted to be “even and uniform”. Another series, involving 50 Thai and Vietnamese patients dying of severe malaria, used thin smears of brain tissue to demonstrate a wide variation in the numbers of parasitized erythrocytes in the individual capillaries within each brain [16]. In several of the brains from the Thai-Vietnam series, some capillaries contained only unparasitized red cells, while others in the same sections had 100% of red cells parasitized. In both these studies, the stages of development varied considerably from vessel to vessel. This study attempts to develop a method that could produce reliable and consistent counts of parasites in brain capillaries, even if their distributions were not uniform. In order to test the hypothesis that the sequestration of late-stage malaria parasites is responsible for the mortality of cerebral malaria, it was important that this method also distinguish parasites of different stages. Stages were chosen that could be recognized on routine histological sections. They were distinguished by the presence or absence of malaria pigment and by its location (intra- *vs* extra-erythrocytic). Young parasites have no pigment and are the most difficult (along with fibrin) to ascertain. Malaria pigment is unambiguously present within infected erythrocytes after 30–34 hours of the 48-hour *P falciparum* life cycle [17], and extra-erythrocytic pigment reflects preceding rupture of mature schizonts. The method involves assessing the proportion of cross-sectioned capillaries containing parasitized erythrocytes and quantifying the stages by counting UPP, PP and PG.

Of the four measures assessed, the traditional measure, %VP, was most reliable. This measure includes both UPP and PP, and discrepancies in their identification and quantification would be absorbed in the more general measure of %VP. This may have contributed to the enhanced reliability of %VP. The least reliable measures were fibrin and UPP. Smaller studies of fibrin by special stain have demonstrated that CM2 can have ~20% of vessels containing fibrin while CM1 shows ~8% of cases with fibrin (Samuel C Wassmer, pers comm). The current validation data appears to have less fibrin than the training data and suggests that studies of fibrin should always include a special stain for fibrin (rather than relying on the appearance of fibrin as stained by haematoxylin and eosin). Similarly, polarized light is a very useful tool for quantifying pigment of any type. Upon expert review, it was clear in the data that UPP were underestimated in the training data by one observer and, thus, this element was used in the repeat CART analysis (all counts by second observer). For researchers employing this method, the use of a superior light source in combination with extensive training may be required for reliable measurement.

The method was used to compare the distribution of all parasites elements in three different areas of the brain. Although the cerebellum appears to be a site of slightly higher overall sequestration, no statistically significant differences in the distribution of parasite elements were observed after correction for multiple testing.

## Conclusions

A method was developed, validated and applied for determining the intensity and distribution of malaria parasites by life-cycle stage in brain capillaries. Consistent observations are possible, especially for the percentage of vessels parasitized. These methods can be used on the usual formalin-fixed, paraffin-embedded, haematoxylin and eosin-stained tissue, and would allow for more standardized comparisons between different autopsy series. This approach can be used for more detailed studies of the intensity and stage distribution of malaria parasites within and between Malaŵian children dying of falciparum malaria.

## Abbreviations

%VP: Percentage of vessels parasitized; CART: Classification and regression tree; Clin CM: Clinical cerebral malaria by WHO definition; CM1: Children meeting the clinical case definition who have predominantly sequestration only in their brains at autopsy; CM2: Children meeting the clinical case definition who have sequestration and intra and extravascular pathology in their brains at autopsy; CM3: Children meeting the clinical case definition who have no evidence of malarial pathology and another anatomic cause of death; NM Coma: Children in a coma with a non-malarial cause; NM Coma IP: Children in a coma with a non-malaria cause and incidental peripheral parasitemia; Other: Children not meeting definitions of CM or NM Coma; PG: Pigment globules; PP: Pigmented parasites; TPG: Total pigment globules; SMA: Severe malaria anaemia; UPP: Unpigmented parasites.

## Competing interests

The authors declare that they have no competing interests.

## Authors’ contributions

DAM designed the study, performed the autopsies, performed the histology experiments, analysed the data, and wrote the manuscript. CV analysed the data and wrote the manuscript. RAC designed the study, performed the autopsies, and performed the histology and electron microscopy experiments. SK and RW performed the autopsies and performed the histology experiments. PBC, NGF and KP performed the histology experiments. KBS, MEM and TET designed the study, recruited the patient population, and wrote the manuscript. All authors read and approved the final manuscript.

## Supplementary Material

Additional file 1 A demonstration of the counting methods used are presented graphically as a slideshow with notes below each slide explaining the method. Click here for file

Additional file 2 **A Bland-Altman difference plot for the % of vessels parasitized (%VP) between two observers (NF and DM) across the first 50 cases.** The red line marks the mean and the blue lines mark 1 standard deviation. Click here for file

Additional file 3 The graphical correlations between NF and DAM for all parasite elements are demonstrated and show that % of vessels parasites, total parasites, and total pigment globules were well correlate based on Spearman correlations, slope, and p-value. Click here for file

Additional file 4 **A comparison of the counts for each parasite element made by NF and DAM across all diagnostic categories (non-collapsed) is presented by element.** Bonferroni and Sidak corrections for the 70 comparisons sets the p-value at less than or equal 0.00074 for significance. For this level, the counts for fibrin (overall) and pigment globules (overall and for CM2) were significantly different with NF > DM for both measures. Click here for file

Additional file 5 **Additional examples of how different elements are distributed across different diagnoses including total pigment, total parasites, and fibrin based on counts by PC for six brain sites across 28 patients.** Data are shown as stacked totals across all sites. In these figures, “non-CM” includes the single CM3 patient in the set of 28. Click here for file

Additional file 6 **The quantitative distribution of all parasite elements across the first 50 case by overall measure and by each diagnosis which demonstrates that, overall, fibrin appears to be more common in the parietal lobe (p-value 0.0227); however, with an appropriate p-value correction (by Bonferroni or Sidak), only values of 0.0014 or less should truly be significant in this data set.** At the individual diagnosis level, there were no differences across sites within an individual element. Click here for file

## References

[B1] WHO Severe falciparum malaria Trans R Soc Trop Med Hyg 2000 94 1 9010748883

[B2] Marchiafava F Bignami: Sulle febbri malariche estivo-autunnali Atti del R Accad Med di Roma 1892 16 291 348

[B3] Dudgeon LS Clarke C An investigation of fatal cases of pernicious malaria caused by *Plasmodium falciparum* in Macedonia Quart J Med 1919 12 372 390

[B4] Edington GM Cerebral malaria in the Gold Coast African four autopsy reports Ann Trop Med Parasitol 1954 48 300 306 13208158 10.1080/00034983.1954.11685627

[B5] Silamut K White NJ Relation of the stage of parasite development in the peripheral blood to prognosis in severe falciparum malaria Trans R Soc Trop Med Hyg 1993 87 436 443 8249075 10.1016/0035-9203(93)90028-o

[B6] Spitz S The pathology of acute falciparum malaria Mil Surg 1946 99 555 572 20276800

[B7] Miller LH Baruch DI Marsh K Doumbo OK The pathogenic basis of malaria Nature 2002 415 673 679 11832955 10.1038/415673a

[B8] Ponsford MJ Medana IM Prapansilp P Hien TT Lee SJ Dondorp AM Esiri MM Day NP White NJ Turner GD Sequestration and microvascular congestion are associated with coma in human cerebral malaria J Infect Dis 2012 205 663 671 22207648 10.1093/infdis/jir812 PMC3266137

[B9] Macpherson GG Warrell MJ White NJ Looareesuwa S Warrell DA Human cerebral malaria: a quantitative ultrastructural analysis of parasitized erythrocyte sequestration Amer J Pathol 1985 119 385 401 3893148 PMC1888001

[B10] Maneerat Y Pongponratn E Viriyavejakul P Punpoowong B Looareesuwan S Udomsangpetch R Cytokines associated with pathology in the brain tissue of fatal malaria Southeast Asian J Trop Med Public Health 1999 30 643 649 10928354

[B11] Nagatake T Hoang VT Tegoshi T Rabbege J Ann TK Aikawa M Pathology of falciparum malaria in Vietnam AmJTrop Med Hyg 1992 47 259 264 10.4269/ajtmh.1992.47.259 1503193

[B12] Pongponratn E Riganti M Punpoowong B Aikawa M Microvascular sequestration of parasitized erythrocytes in human falciparum malaria: a pathological study AmJTrop Med Hyg 1991 44 168 175 10.4269/ajtmh.1991.44.168 2012260

[B13] Riganti M Pongponratn E Tegoshi T Looareesuwa S Punpoowong B Aikawa M Human cerebral malaria in Thailand: a clinico-pathological correlation Immunol Lett 1990 25 199 206 2283150 10.1016/0165-2478(90)90115-7

[B14] Molyneux M Taylor T Wirima J Borgstein A Clinical features and prognostic indicators in paediatric cerebral malaria: a study of 131 comatose Malawian children Quart J Med 1989 71 441 459 2690177

[B15] Taylor T Fu W Carr R Whitten R Mueller J Fosiko N Lewallen S Liomba N Molyneux M Differentiating the pathologies of cerebral malaria by postmortem parasite counts Nat Med 2004 10 143 145 14745442 10.1038/nm986

[B16] McCullagh P Nealder F Generalized Linear Models 1989 2 New York: Chapman and Hall

[B17] Silamut K Phu NH Whitty C Turner GD Louwrier K Mai NT Simpson JA Hien TT White NJ A quantitative analysis of the microvascular sequestration of malaria parasites in the human brain Am J Pathol 1999 155 395 410 10433933 10.1016/S0002-9440(10)65136-X PMC1866852

