# Supplementary material for: A histological method for quantifying Plasmodium falciparum in the brain in fatal paediatric cerebral malaria
Source: Malar J. 2013 Jun 7;12:191. doi: 10.1186/1475-2875-12-191 (PMC3701562; doi:10.1186/1475-2875-12-191)
Supplement: Additional file 1 — A demonstration of the counting methods used are presented graphically as a slideshow with notes below each slide explaining the method. [file 1475-2875-12-191-S1.ppt]

## Slide 1
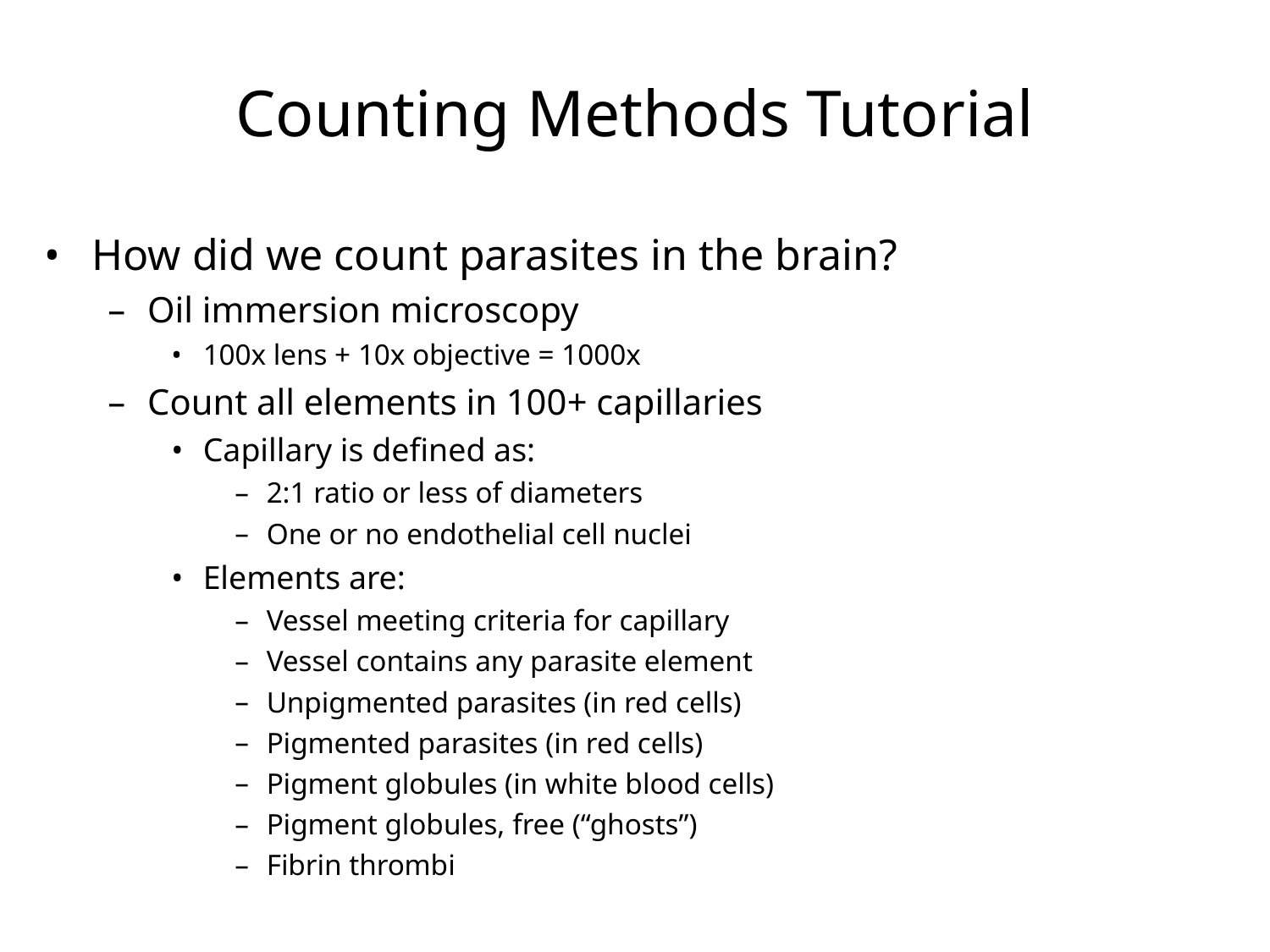

# Counting Methods Tutorial
How did we count parasites in the brain?
Oil immersion microscopy
100x lens + 10x objective = 1000x
Count all elements in 100+ capillaries
Capillary is defined as:
2:1 ratio or less of diameters
One or no endothelial cell nuclei
Elements are:
Vessel meeting criteria for capillary
Vessel contains any parasite element
Unpigmented parasites (in red cells)
Pigmented parasites (in red cells)
Pigment globules (in white blood cells)
Pigment globules, free (“ghosts”)
Fibrin thrombi

## Slide 2
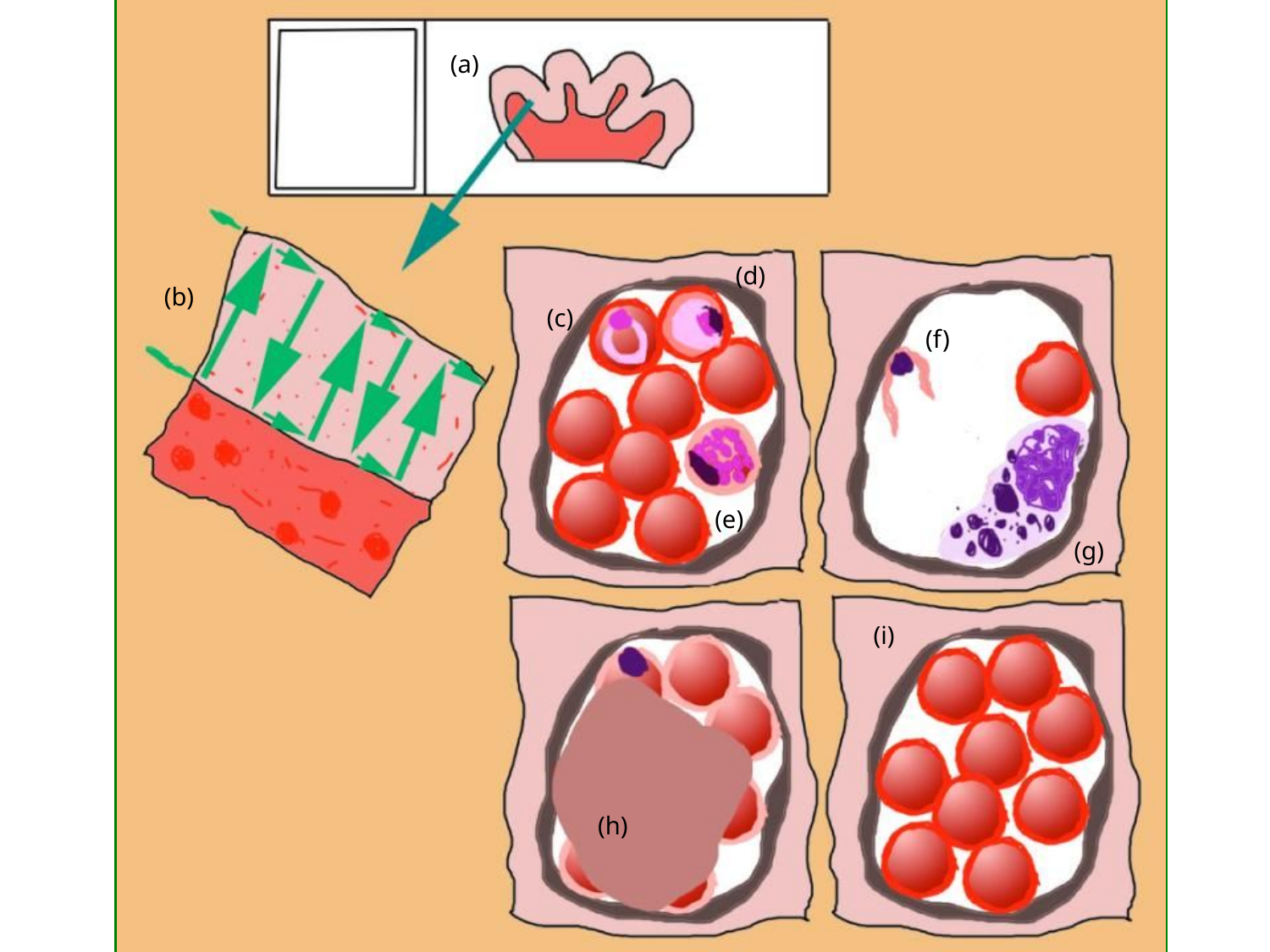

(a)
(d)
(b)
(c)
(f)
(e)
(g)
(i)
(h)

## Slide 3
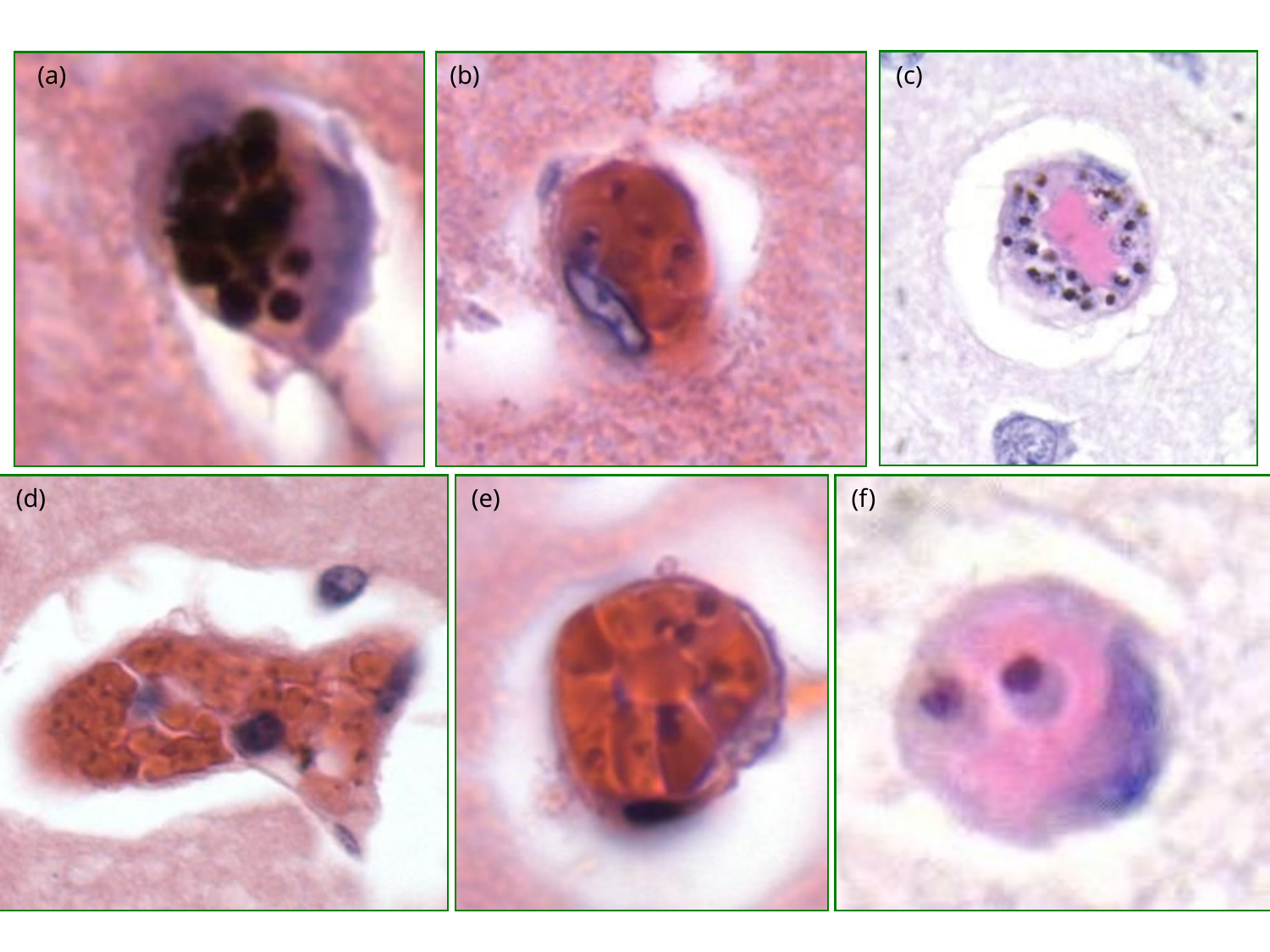

(a)
(b)
(c)
(d)
(e)
(f)

## Slide 4
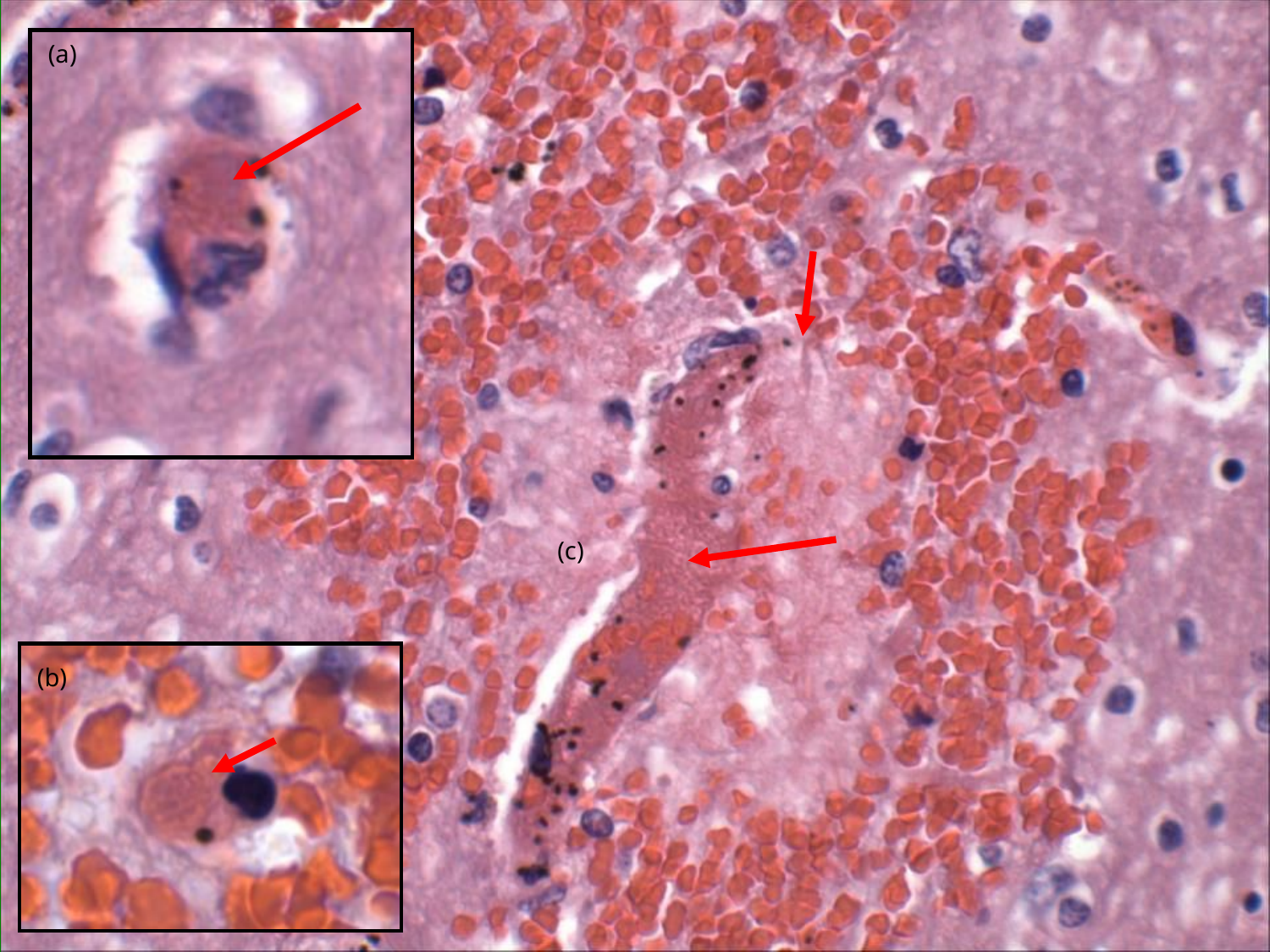

(a)
(c)
(b)
