# Supplementary figures and images for: A histological method for quantifying Plasmodium falciparum in the brain in fatal paediatric cerebral malaria
Source: Malar J. 2013 Jun 7;12:191. doi: 10.1186/1475-2875-12-191 (PMC3701562; doi:10.1186/1475-2875-12-191)

**% of Vessels Parasitized (Bland-Altman Plot)**

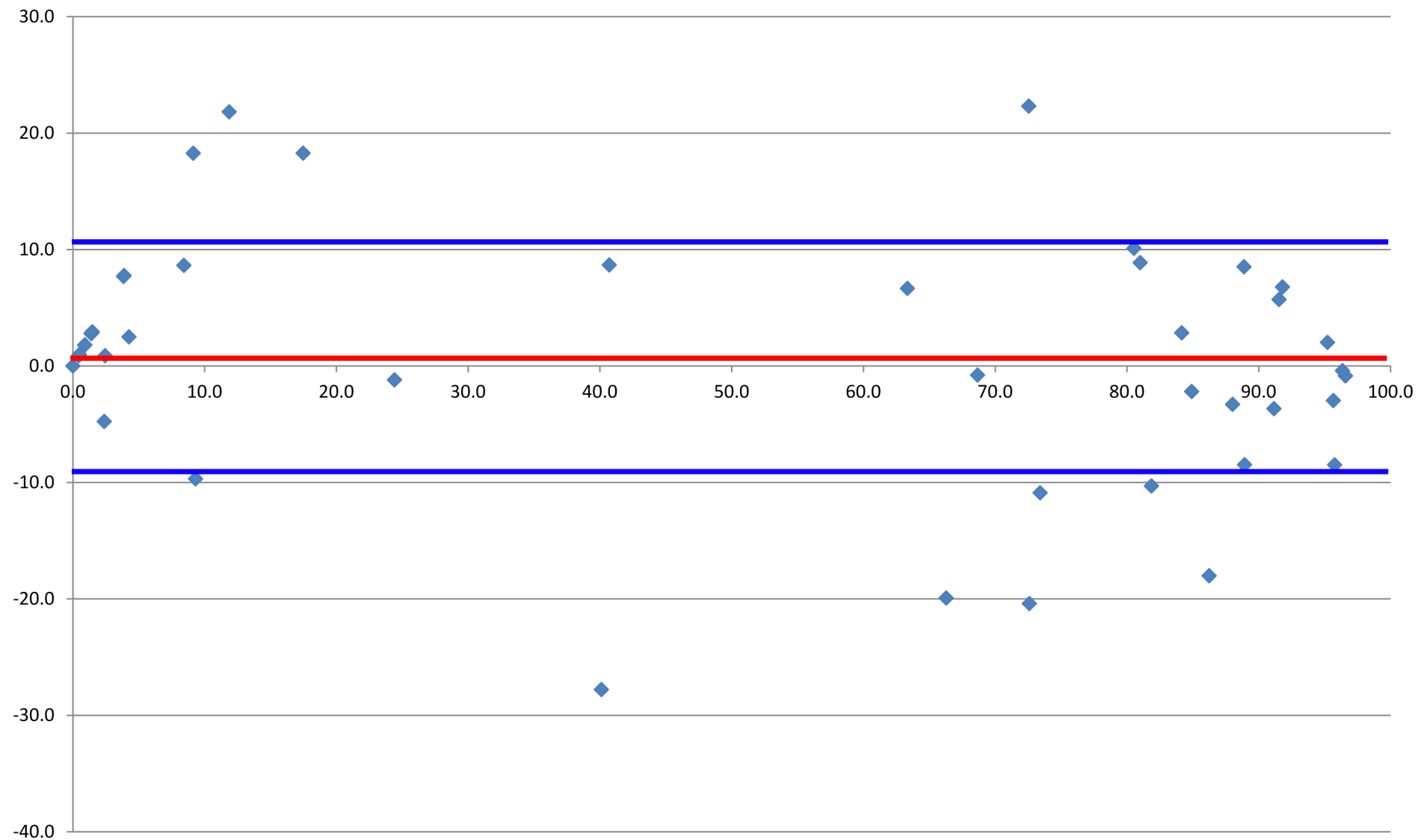

Supplement: Additional file 2 — A Bland-Altman difference plot for the % of vessels parasitized (%VP) between two observers (NF and DM) across the first 50 cases. The red line marks the mean and the blue lines mark 1 standard deviation. [file 1475-2875-12-191-S2.pdf]

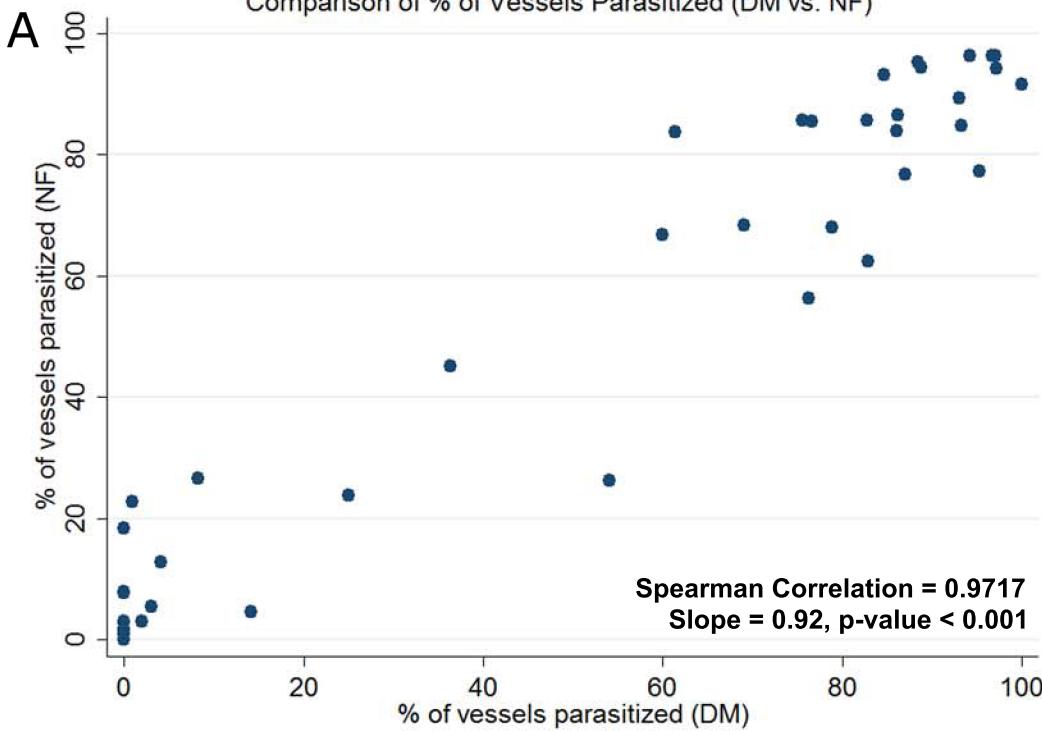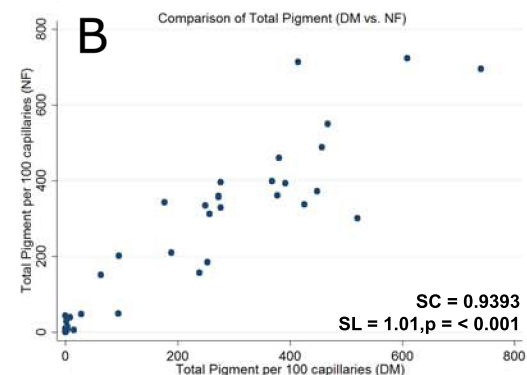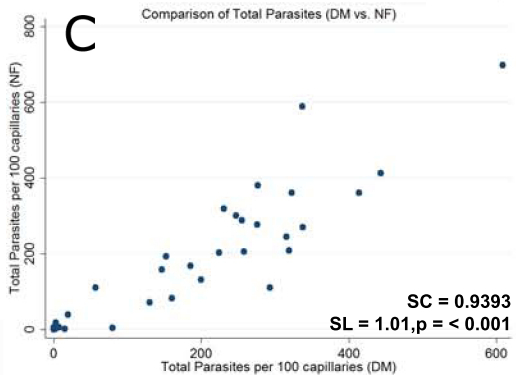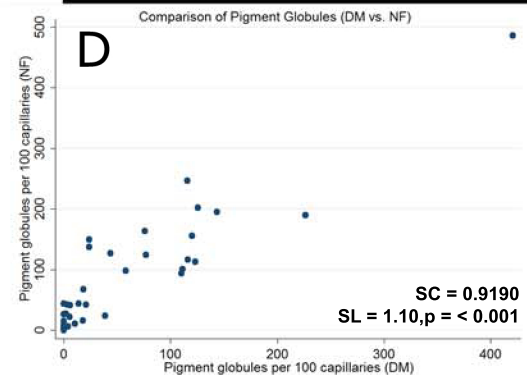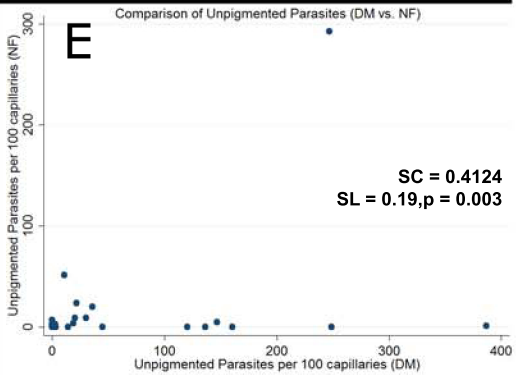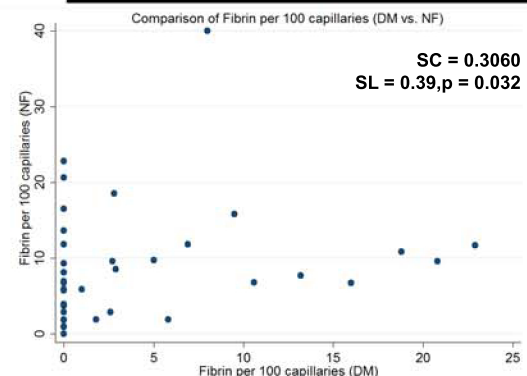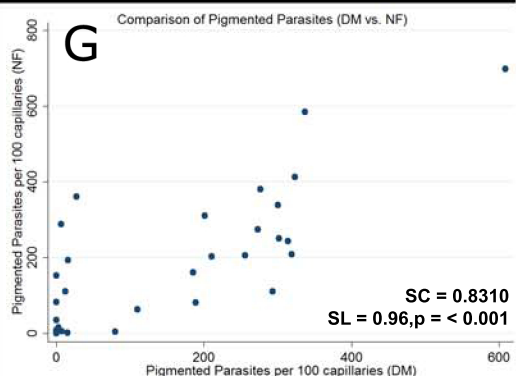

Supplement: Additional file 3 — The graphical correlations between NF and DAM for all parasite elements are demonstrated and show that % of vessels parasites, total parasites, and total pigment globules were well correlate based on Spearman correlations, slope, and p-value. [file 1475-2875-12-191-S3.pdf]

## Slide 1
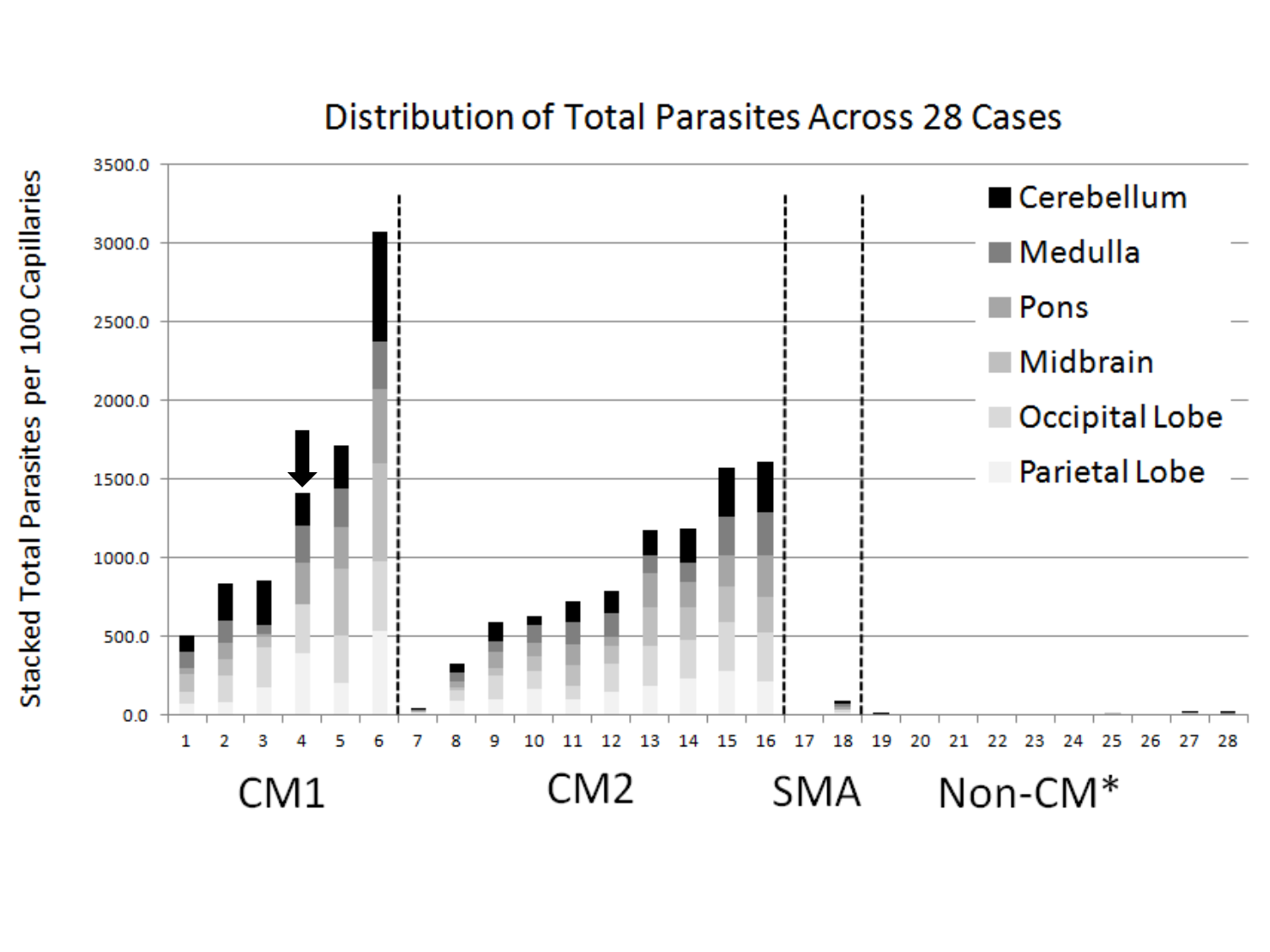

## Slide 2
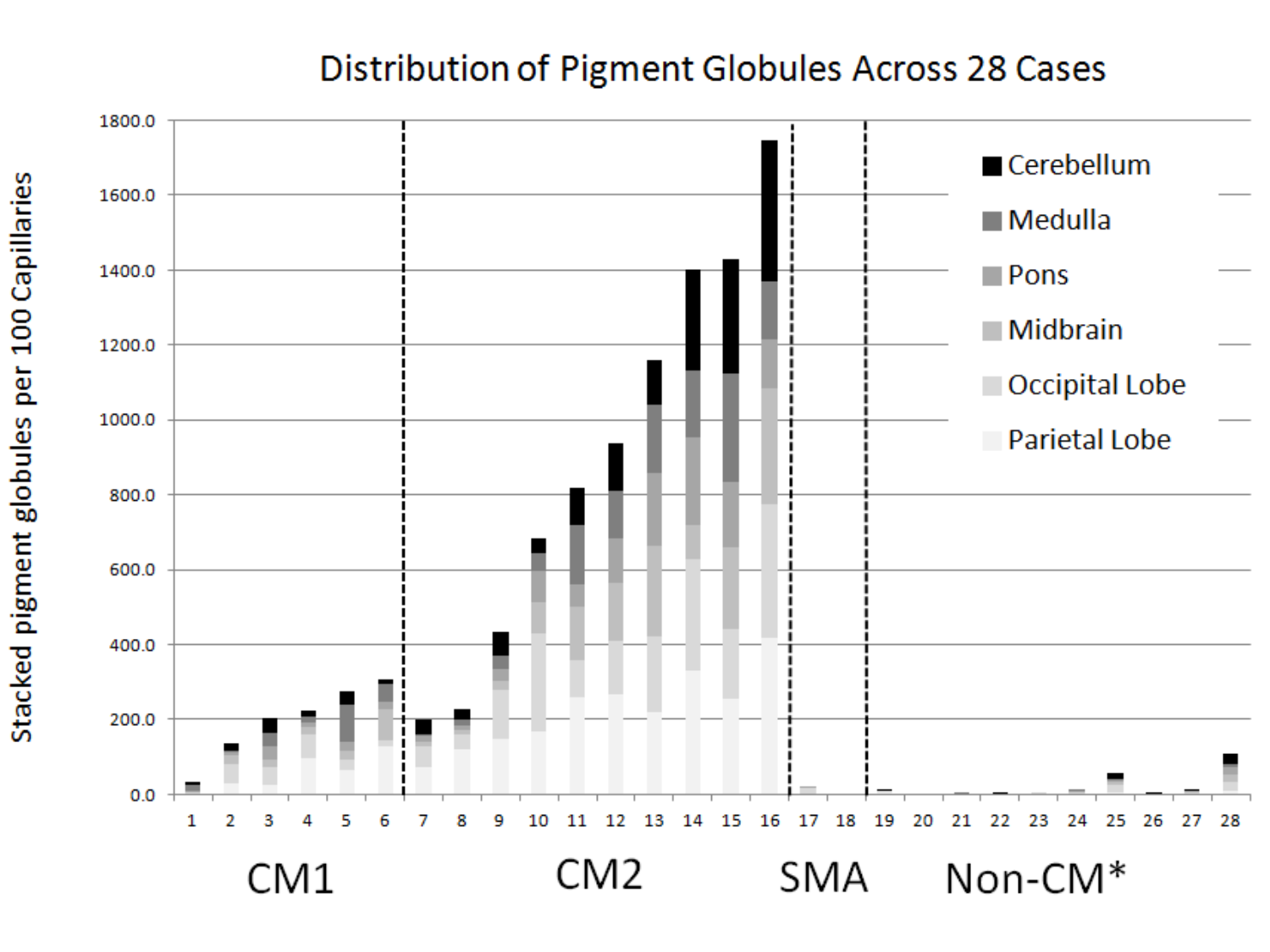

## Slide 3
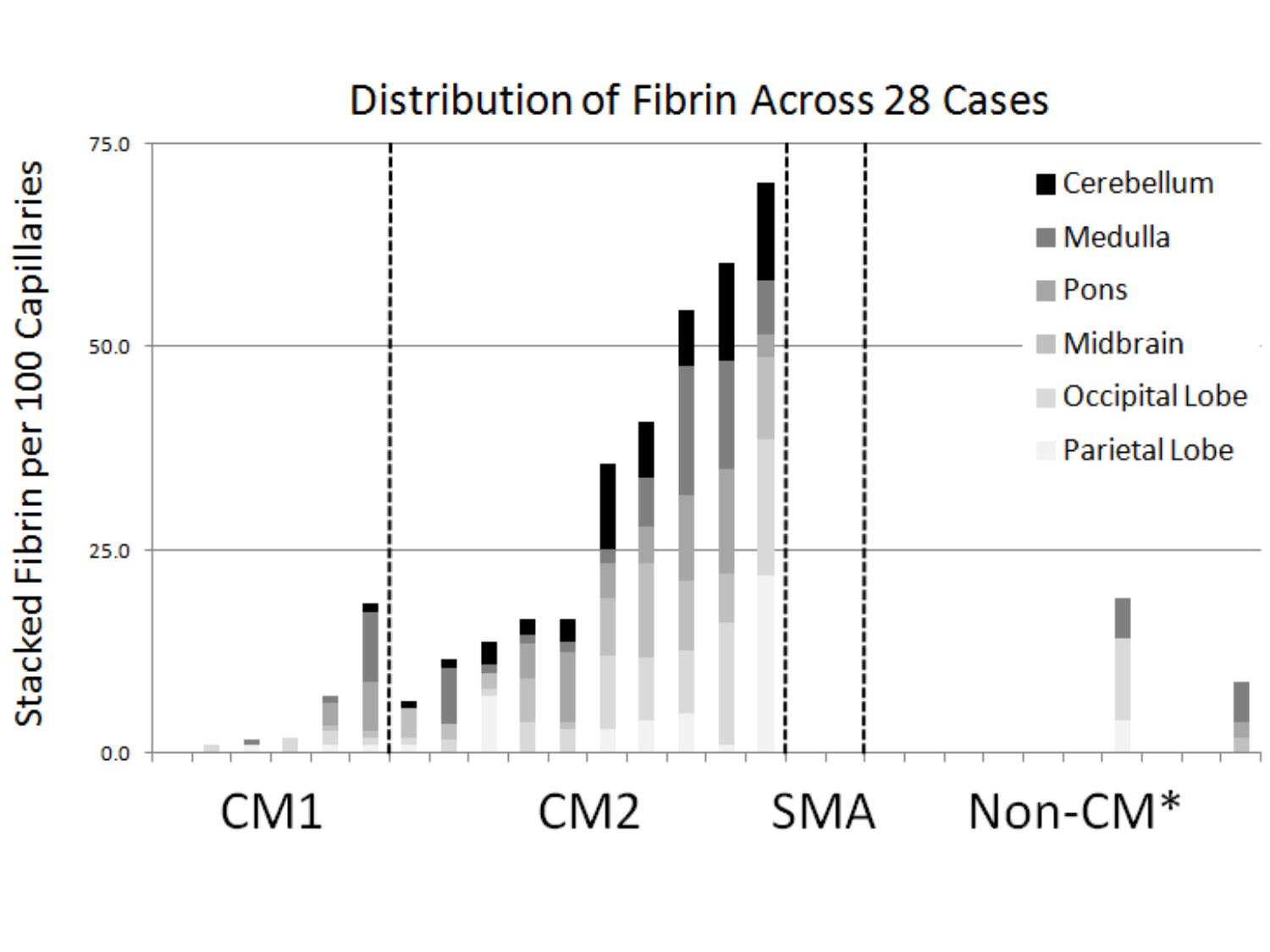

Supplement: Additional file 5 — Additional examples of how different elements are distributed across different diagnoses including total pigment, total parasites, and fibrin based on counts by PC for six brain sites across 28 patients. Data are shown as stacked totals across all sites. In these figures, “non-CM” includes the single CM3 patient in the set of 28. [file 1475-2875-12-191-S5.ppt]
